# Inhibition of African swine fever virus in liquid and feed by medium-chain fatty acids and glycerol monolaurate

**DOI:** 10.1186/s40104-020-00517-3

**Published:** 2020-12-08

**Authors:** Joshua A. Jackman, Astghik Hakobyan, Hovakim Zakaryan, Charles C. Elrod

**Affiliations:** 1 School of Chemical Engineering, Sungkyunkwan University, Suwon, 16419 Republic of Korea; 2 Group of Antiviral Defense Mechanisms, Institute of Molecular Biology of NAS, Yerevan, Armenia; 3 Natural Biologics Inc., Newfield, NY 14867 USA; 4 Department of Animal Science, Cornell University, Ithaca, NY 14853 USA

**Keywords:** African swine fever virus, Antiviral, Feed pathogen mitigation, MCFA, Medium-chain fatty acids, Monoglycerides

## Abstract

**Background:**

The ongoing African swine fever virus (ASFv) epidemic has had a major impact on pig production globally and biosecurity efforts to curb ASFv infectivity and transmission are a high priority. It has been recently identified that feed and feed ingredients, along with drinking water, can serve as transmission vehicles and might facilitate transboundary spread of ASFv. Thus, it is important to test the antiviral activity of regulatory compatible, antiviral feed additives that might inhibit ASFv infectivity in feed. One promising group of feed additive candidates includes medium-chain fatty acids (MCFA) and monoglyceride derivatives, which are known to disrupt the lipid membrane surrounding certain enveloped viruses and bacteria.

**Results:**

The antiviral activities of selected MCFA, namely caprylic, capric, and lauric acids, and a related monoglyceride, glycerol monolaurate (GML), to inhibit ASFv in liquid and feed conditions were investigated and suitable compounds and inclusion rates were identified that might be useful for mitigating ASFv in feed environments. Antiviral assays showed that all tested MCFA and GML inhibit ASFv. GML was more potent than MCFA because it worked at a lower concentration and inhibited ASFv due to direct virucidal activity along with one or more other antiviral mechanisms. Dose-dependent feed experiments further showed that sufficiently high GML doses can significantly reduce ASFv infectivity in feed in a linear manner in periods as short as 30 min, as determined by infectious viral titer measurements. Enzyme-linked immunosorbent assay (ELISA) experiments revealed that GML treatment also hinders antibody recognition of the membrane-associated ASFv p72 structural protein, which likely relates to protein conformational changes arising from viral membrane disruption.

**Conclusion:**

Together, the findings in this study indicate that MCFA and GML inhibit ASFv in liquid conditions and that GML is also able to reduce ASFv infectivity in feed, which may help to curb disease transmission.

**Supplementary information:**

The online version contains supplementary material available at 10.1186/s40104-020-00517-3.

## Introduction

The African swine fever virus (ASFv) causes lethal disease in pigs with mortality rates that can approach 100% in naïve pig populations [1, 2]. Over the past few years, the spread of ASFv has reached epidemic levels, and a large fraction of the pig population in many nations, especially in East Asia, has died from the disease or been culled to ward off disease spread [3, 4]. Accordingly, the ongoing ASFv epidemic is having severe economic consequences for global food and feed markets [5, 6]. Currently, there are no approved vaccines or therapeutics for ASFv [7], and thus there is an urgent need for increased biosecurity efforts to curb ASFv infectivity and transmission.

Among factors contributing to disease spread, there is growing attention to the role of feed and feed ingredients, which are regarded as a moderate-risk transmission vehicle for ASFv and other swine viral pathogens [8–12]. Niederwerder et al. showed that ASFv can be transmitted orally in liquids and in plant-based feed [13], which fits with epidemiological findings linking feed transmission to a past ASFv outbreak in Latvia [14]. Such findings motivate the development of mitigation strategies to reduce the infectivity of virus-contaminated feed and thus decrease feed-mediated infection probability, i.e., suppress growth of virus particle and infected cell populations in pigs [15]. One of the first swine viruses associated with feed transmission was porcine epidemic diarrhea virus (PEDv) [16–18] and a formaldehyde-based additive has been demonstrated to reduce PEDv infectivity in feed [19, 20]. While formaldehyde is approved as a swine feed additive in the United States, it is banned as a feed additive in some jurisdictions such as the European Union [Regulation (EU) 2018/183], which has led to the exploration of other regulatory acceptable feed additives with potential antiviral properties. In this regard, one promising class of feed additives consists of medium-chain fatty acids (MCFA), which are 6–12 carbon long saturated fatty acids. MCFA are receiving attention because they can inhibit a wide range of membrane-enveloped viruses and bacteria through membrane-disruptive effects and have also been shown to support growth performance in pig production [21, 22]. The main MCFA are 6-carbon caproic, 8-carbon caprylic, 10-carbon capric, and 12-carbon lauric acids.

Early work demonstrated that an MCFA blend (1:1:1 ratio of caproic, caprylic, and capric acids) was as effective as formaldehyde at inhibiting PEDv in feed [23], and similar performance results were also obtained with *Salmonella* Typhimurium in feed [24]. More systematic studies [25, 26] aimed at defining inclusion rate and MCFA composition have been performed and demonstrated that caproic, caprylic, and capric acids individually mitigated PEDv in feed to similar extents as formaldehyde. Within this scope, there has also been limited exploration of glycerol monolaurate (GML), which is the monoglyceride derivative of lauric acid that is often regarded as more biologically potent than MCFA *in vitro* [27], as a component within a commercial feed additive product [26] and as part of a blend comprising MCFA, GML, and lactic acid [28]. To date, the main focus of exploring MCFA and GML as potential antiviral feed additives to inhibit swine viral pathogens has been PEDv mitigation. Notably, those past studies relied on indirect readouts of antiviral effects, including polymerase chain reaction (PCR) testing to detect viral nucleic acids and an *in vivo* pig bioassay to evaluate viral transmissibility. There remains an outstanding need to investigate the extent to which different MCFA and GML might inhibit ASFv, and to quantitatively measure how much these feed additive candidates affect viral infectivity based on direct virological readouts.

Herein, the antiviral activities of selected MCFA, namely caprylic, capric, and lauric acids, along with GML to inhibit ASFv in liquid and feed conditions were investigated, and suitable compounds and inclusion rates were identified that might be useful for mitigating ASFv in feed environments. Antiviral experiments in liquid conditions screened the extent of virucidal activity among the test compounds along with more detailed evaluation of selected compounds. Dose-dependent feed experiments were also conducted to measure how feed additive candidates, a blended MCFA mixture or GML, affected ASFv infectivity in feed, as determined by infectious viral titer measurements. Additional PCR and enzyme-linked immunosorbent assay (ELISA) experiments provided mechanistic insight into ASFv inhibition in feed.

## Methods

### Reagents

Caprylic acid (PHR1202-1G), capric acid (C1875-100G), lauric acid (W261408-1KG-K), and glycerol monolaurate (M1765-100MG) were obtained from Sigma-Aldrich (Schnelldorf, Germany) and used for antiviral testing in solution. For the feed experiments, two dry feed additive candidates, (1) an MCFA blend containing caprylic, capric, and lauric acids (51:29:7 ratio) plus silica carrier and (2) GML, were provided by Natural Biologics Inc. (New York, USA). The MCFA-based feed additive is a laboratory-scale prototype mixture that contains 65% total MCFA and 35% silica. The MCFA blend is liquid at room temperature and was applied to the silica carrier at room temperature, followed by mixing until the sample was uniform, dry, and free flowing.

### Virus and cell culture

The ASFv BA71V strain was used for all experiments and is adapted to grow in Vero cells of African green monkey kidney origin [29]. Virus titration was performed by a cytopathic effect (CPE) assay in Vero cells in a 10-fold serial dilution format, as previously described [30]. The viral titer was measured by the Spearman-Kärber endpoint method and reported as log 50% tissue culture infective dose (TCID_50_) per mL (log TCID_50_/mL). Vero cells were used for all experiments, and were maintained and propagated at 37 °C in Eagle’s minimum essential medium (EMEM) (Lonza, Belgium) that was supplemented with 10% fetal bovine serum (FBS), 2 mmol/L *L*-glutamine, 100 international-units/mL penicillin, and 100 μg/mL streptomycin (Sigma-Aldrich, Germany), as previously described [30].

### Virucidal assay in solution

A virus suspension containing 1.6 × 10^5^ TCID_50_ ASFv particles per well was incubated with test compounds for 1 h at room temperature. Then, the treated virus suspension was diluted 20-fold before adding the diluted suspension to infect 2 × 10^4^ Vero cells in a 96-well cell culture plate. After 1-h incubation at 37 °C, the cells were extensively washed with phosphate-buffered saline (PBS) and then EMEM supplemented with 3% FBS was added. Cell infection was allowed to proceed until complete CPE was developed in virus-only control wells (usually 3–4 d post-infection). Then, the cell supernatants were collected and viral titers in the supernatants were determined by a CPE-based titration assay. Serial dilutions of the supernatants were prepared and inoculated onto Vero cells in a 96-well cell culture plate (6 wells per dilution). The number of wells that were infected was then determined for each virus dilution, and the viral titer was measured by the Spearman-Kärber method.

### Antiviral assay in solution

A virus suspension (multiplicity of infection: 0.2 TCID_50_ per cell) was incubated with test compounds for 1 h at room temperature. Then, the virus-compound mixtures were added to Vero cells seeded at 2 × 10^5^ cells per well in a 24-well culture plate (4 wells per sample). Cell infection was allowed to proceed until complete CPE was developed in virus-only control wells (usually 3–4 d post-infection). Then, the cell supernatants were collected and viral titers in the supernatants were determined by a CPE-based titration assay as described above.

### Feed sample preparation

A powdered mixture of MCFA or GML was used as the active ingredient and suspended in EMEM. Two grams of commercial swine feed (Aktifeed Grow 20%, Mustang Nutrition Technology, Moscow, Russia) were mixed with the active ingredient by vortexing at inclusion rates of 0.25%, 0.50%, 1.0%, or 2.0% (wt/wt). Then, each feed sample was incubated with 100 μL of EMEM containing ASFv at 10^6^ TCID_50_ dosage for 30 min or 24 h at room temperature. After incubation, 20 mL of fresh EMEM was added to each feed sample and centrifuged at 3600×*g* for 40 min (4 °C). The supernatant was collected, and aliquots were analyzed in virus recovery, PCR, and ELISA tests. Positive control (virus-only) samples were incubated with the same amount of virus without active ingredients.

### Virus recovery assay of feed samples

90% confluent Vero cells in a 96-well culture plate were inoculated with the supernatant diluted in a 10-fold series (6 wells per dilution). After 96 h, viral titers were determined as described above.

### PCR analysis of feed samples

Total DNA was extracted using the DNeasy Blood & Tissue kit (Qiagen, Germany). The real-time PCR method and primers for detecting the ASFv p72 gene were used in accordance with the recommendations of the World Organization for Animal Health [31, 32]. Positive amplification controls consisted of DNA extracted from ASFv virus stock and DNA extracted from virus-only feed samples. Negative amplification controls consisted of DNA extracted from mock-infected Vero cells. The cycle threshold (Ct) of each sample was measured.

### ELISA analysis of feed samples

The ASFv p72 protein level was measured by using a commercially available ELISA kit (INgezim PPA DAS, 3465RD, Ingenasa, Madrid, Spain) and a colorimetric reader, as previously described [33]. The optical density (OD) of each sample was measured at 405 nm wavelength.

### Statistical analysis

All statistical tests were evaluated by using the GraphPad Prism software package (California, USA). One-way analysis of variance (ANOVA) with Dunnett’s multiple comparisons test (versus virus-only positive control; unless otherwise specified), the unpaired Student’s t-test (versus virus-only positive control), or linear regression analysis (dose-dependent effects) was used depending on the experiment. Statistical significance was computed in terms of standard or multiplicity-adjusted *P* values, and *P* < 0.05, *P* < 0.01, and *P* < 0.001 indicate the levels of statistical significance (*, **, ***).

## Results and discussion

### Antiviral activity in aqueous solution

**Fig. 1:**
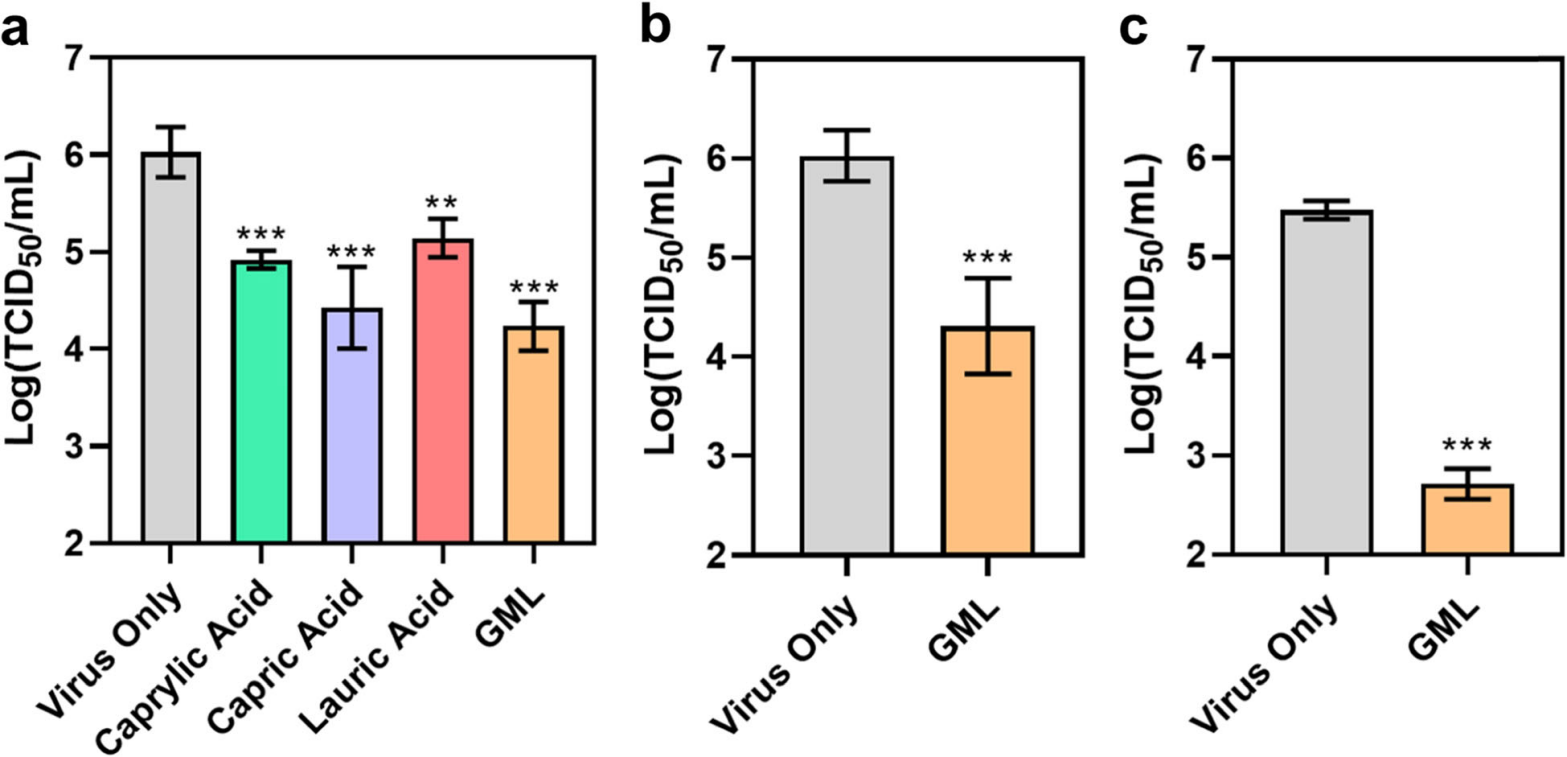
Evaluation of MCFA and GML to inhibit ASFv infectivity in aqueous solution. **a** Virucidal screening assay to determine inhibitory effect on extracellular ASFv particles. The compound concentration was 5 mmol/L. Infectious viral titers were measured by CPE-based assay (*n* = 6 in virus-only control and *n* = 3 in other groups). **b** Virucidal assay results for 250 μmol/L GML treatment (*n* = 6 in virus-only control and *n* = 3 in GML-treated group). Same method as panel (**a**). **c** Antiviral assay results for 250 μmol/L GML treatment (*n* = 3 per group). Viral titers were determined by CPE-based assay. In panels (**a**)-(**c**), data are reported as mean ± standard deviation. The markers ** and *** indicate *P* < 0.01 and *P* < 0.001, respectively, versus the virus-only control

ASFv is genetically distinct from other swine viral pathogens and is the only member of the Asfarviridae family [34]. A particularly unique feature of ASFv particles is that they have a complex structure that includes two distinct layers of lipid bilayer coating [35] as opposed to the more conventional one layer in most other membrane-enveloped viruses such as PEDv [36, 37]. Thus, while MCFA and GML are known to inhibit a wide range of membrane-enveloped viruses, focused testing against ASFv and its more complex, double-membrane structure is warranted to evaluate possible antiviral activity and rank potency among different MCFA and GML candidates. Therefore, we first measured the *in vitro* neutralizing activity of the test compounds to abrogate ASFv infectivity in a solution-phase virucidal assay based on direct interaction of the test compounds with membrane-enveloped ASFv particles (Fig. 1a). Virus suspensions in PBS were incubated with one of the test compounds at 5 mmol/L concentration for 1 h at 37 °C, and then the viral titer of each suspension was determined.

The virus-only control had a mean viral titer of 6.03 log TCID_50_/mL while marked reductions in viral infectivity were observed upon treatment with the test compounds. Treatment with caprylic acid or capric acid caused significant decreases in mean viral titer down to 4.92 and 4.42 log TCID_50_/mL, respectively. These changes correspond to greater than 92 and 97% reductions in viral infectivity. Likewise, treatment with lauric acid or GML caused significant decreases in mean viral titer down to 5.14 and 4.32 log TCID_50_/mL, respectively. These changes correspond to greater than 87 and 98% reductions in viral infectivity. Thus, all tested MCFA and GML at 5 mmol/L compound concentrations exhibited virucidal activity and significantly reduced ASFv infectivity compared to the virus-only control.

Based on the high antiviral activity of GML and greater reported *in vitro* potency than MCFA against other enveloped viruses [38, 39], we further examined the virucidal activity of GML to inhibit ASFv at 250 μmol/L concentration and this lower concentration of GML still caused significant drops in viral infectivity (Fig. 1b). In this case, the mean viral titer of the GML-treated group decreased to 4.31 log TCID_50_/mL, which also corresponds to greater than 98% reduction in viral infectivity. The effect of 250 μmol/L GML treatment on ASFv infectivity and replication was also tested in an antiviral assay (Fig. 1c). In this assay, the virus suspension in PBS was incubated with 250 μmol/L GML for 1 h at 37 °C before the virus-GML mixture was directly added to infect Vero cells, followed by viral titer measurements. Notably, the mean viral titer significantly decreased from 5.48 log TCID_50_/mL in the virus-only control to 2.71 log TCID_50_/mL in the 250 μmol/L GML-treated group, which corresponds to a greater than 99.8% reduction in viral infectivity.

Additional antiviral assay experiments at lower GML concentrations showed concentration-dependent inhibitory activity across the range from 250 to 63 μmol/L GML (*P* < 0.001) (Additional file 1: Fig. S1). Treatment with 125 or 63 μmol/L GML caused the mean viral titer to significantly decrease to 3.70 and 4.69 log TCID_50_/mL, respectively. These changes correspond to greater than 98% and 83% reductions in viral infectivity. On the other hand, 31 μmol/L GML and lower concentrations were inactive. Taking into account that the critical micelle concentration – the lowest concentration at which nanostructured assemblies called micelles form – of GML is 60 μmol/L [40, 41], these results support that GML is mainly active in the micellar state as opposed to the monomeric state, which agrees well with membrane biophysics literature reports [42]. Moreover, the GML-induced decreases in viral infectivity in the antiviral assay were appreciably larger than those in the virucidal assay, which further supports that GML not only directly inhibits ASFv particle infectivity but also exhibits additional mechanisms of antiviral activity. For example, it has been reported that GML treatment can stabilize mammalian cell membranes in order to protect against infectious pathogens [43] as well as to regulate cell signaling pathways [44]. Sola et al. have also reported that two longer-chain, unsaturated monoglycerides – monoolein and monolinolein – inhibit ASFv by at least two antiviral mechanisms as well [45]. We also tested the three MCFA at 250 μmol/L in the antiviral activity assay and they were all inactive, indicating that GML exhibits antiviral activity at a lower concentration than the three MCFA (*P* < 0.001) (Additional file 1: Fig. S2). Collectively, our findings demonstrate that GML potently inhibits ASFv in aqueous liquid conditions and can significantly reduce viral infectivity due to virucidal activity and other antiviral mechanisms.

### Antiviral activity in feed

We proceeded to test the effects of MCFA and GML as feed additives to reduce the infectivity of ASFv-contaminated feed. Two types of feed additives were tested: (1) a blended MCFA mixture of caprylic, capric, and lauric acids (51:29:7 ratio plus silica carrier); and (2) GML. The additives were incorporated into the feed at inclusion rates of 0.25%, 0.50%, 1.0%, and 2.0% and then the feed samples were spiked with ASFv inoculums. The viral titer in the feed was measured after a 30-min or 24-h incubation period.

**Fig. 2:**
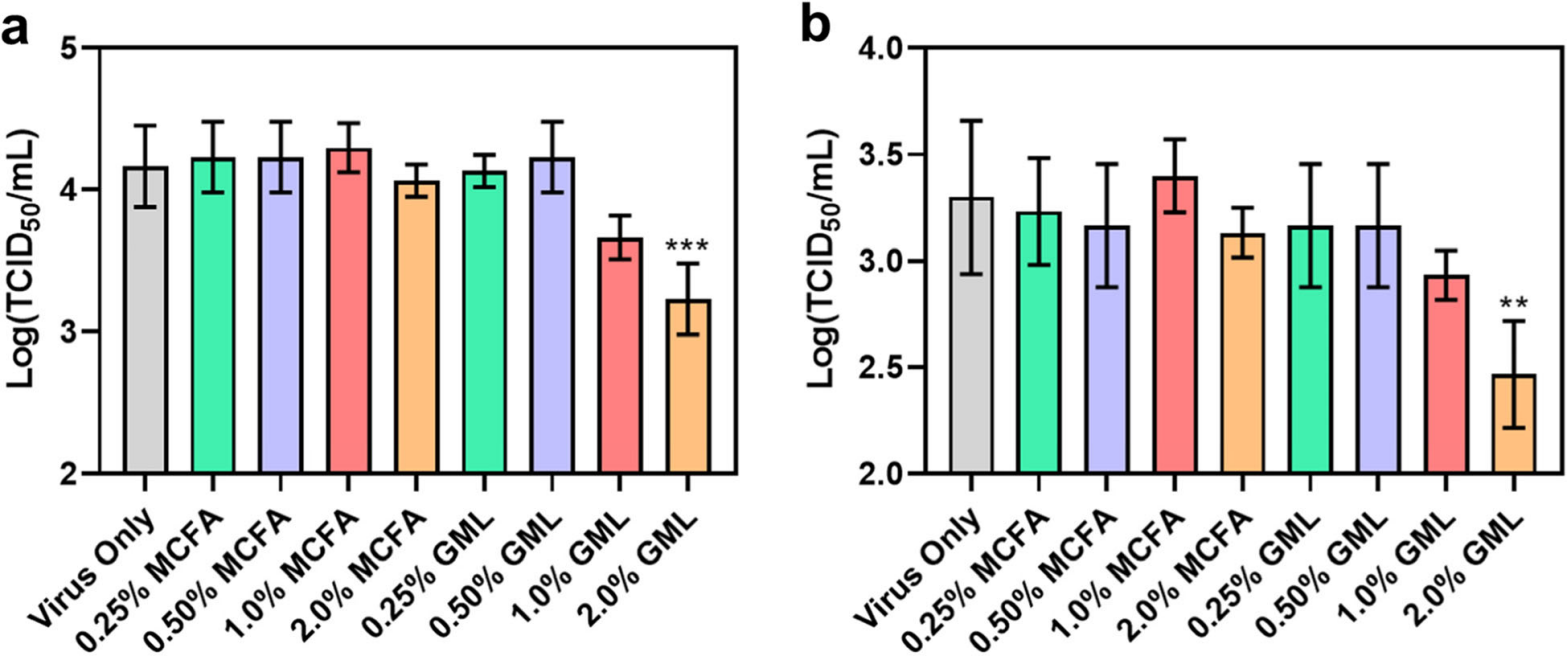
Effect of MCFA mixture and GML additives on ASFv infectivity in feed. 0–2.0% (wt/wt) feed additives were included in the feed and the effects on ASFv infectivity were measured (**a**) 30 min and (**b**) 24 h post-incubation. Viral titers were measured by CPE-based assay. Data are reported as mean ± standard deviation from three independent experiments (*n* = 3 per group). The markers ** and *** indicate *P* < 0.01 and *P* < 0.001, respectively, versus the virus-only control

After 30-min incubation, the virus-only control had a mean viral titer of 4.17 log TCID_50_/mL, while the viral titers of all MCFA-treated feed samples were similar, indicating that the MCFA blend did not reduce viral infectivity (Fig. 2a). In marked contrast, the GML-treated feed samples exhibited dose-dependent reductions in viral infectivity at 1.0% and higher inclusion rates (*P* < 0.01). The mean viral titers of 0.25% and 0.50% GML-treated feed samples were similar to the virus-only control, while the mean viral titer of the 1.0% GML-treated feed sample tended to decrease to 3.67 log TCID_50_/mL, which corresponds to a greater than 68% reduction in viral infectivity. A significant decrease in viral infectivity occurred in the 2.0% GML-treated feed sample and the mean viral titer dropped to 3.23 log TCID_50_/mL, which corresponds to a greater than 88% reduction in viral infectivity. These data show that GML can quickly reduce viral infectivity in ASFv-contaminated feed whereas the MCFA blend was inactive.

We also measured the effect of the two feed additives on viral infectivity after a 24-h incubation period (Fig. 2b). In this case, the mean viral titer of the virus-only control had decreased to 3.30 log TCID_50_/mL, which corresponds to a greater than a 86% drop in viral infectivity compared to the 30-min virus-only control and is consistent with the reported half-lives of around 1.3 to 2.2 d for ASFv in feed matrices [46]. The mean viral titers of the MCFA-treated feed samples were similar to the 24-h virus-only control at all tested doses, while GML exhibited dose-dependent inhibitory effects at 1.0% and higher inclusion rates (*P* < 0.01). The 0.25 and 0.50% GML doses were inactive, whereas the 1.0% GML dose tended to reduce the mean viral titer to 2.93 log TCID_50_/mL, which corresponds to a 57% reduction in viral infectivity compared to the 24-h virus-only control. Moreover, the 2.0% GML dose significantly reduced the mean viral titer to 2.47 log TCID_50_/mL, which corresponds to an 85% reduction in viral infectivity compared to the 24-h virus-only control.

Together, these data show that the GML additive reduces viral infectivity in ASFv-contaminated feed samples. Importantly, the inclusion of 2.0% GML in the feed not only accelerated the inhibition of viral infectivity in the contaminated feed as noted by significant decreases after only 30 min but also led to more pronounced drops in viral infectivity overall. Compared to the 30-min virus control, the 2.0% GML additive reduced viral infectivity by 98% within 24 h, which translates into a much lower infection probability for animals such as pigs that might consume virus-contaminated feed [13].

### Effect on virus genetic material

In addition, quantitative PCR measurements were conducted on the ASFv-contaminated feed samples in order to determine if the MCFA or GML additives affect virus genetic material, which is mainly present within the ASFv particles. The experiments were conducted because virus genetic material is often used as a diagnostic marker for virus contamination in feed and we measured the cycle threshold (Ct) value, which is inversely proportional to the amount of intact viral DNA present within a feed sample. A higher Ct value indicates less intact genetic material and vice versa.

**Fig. 3:**
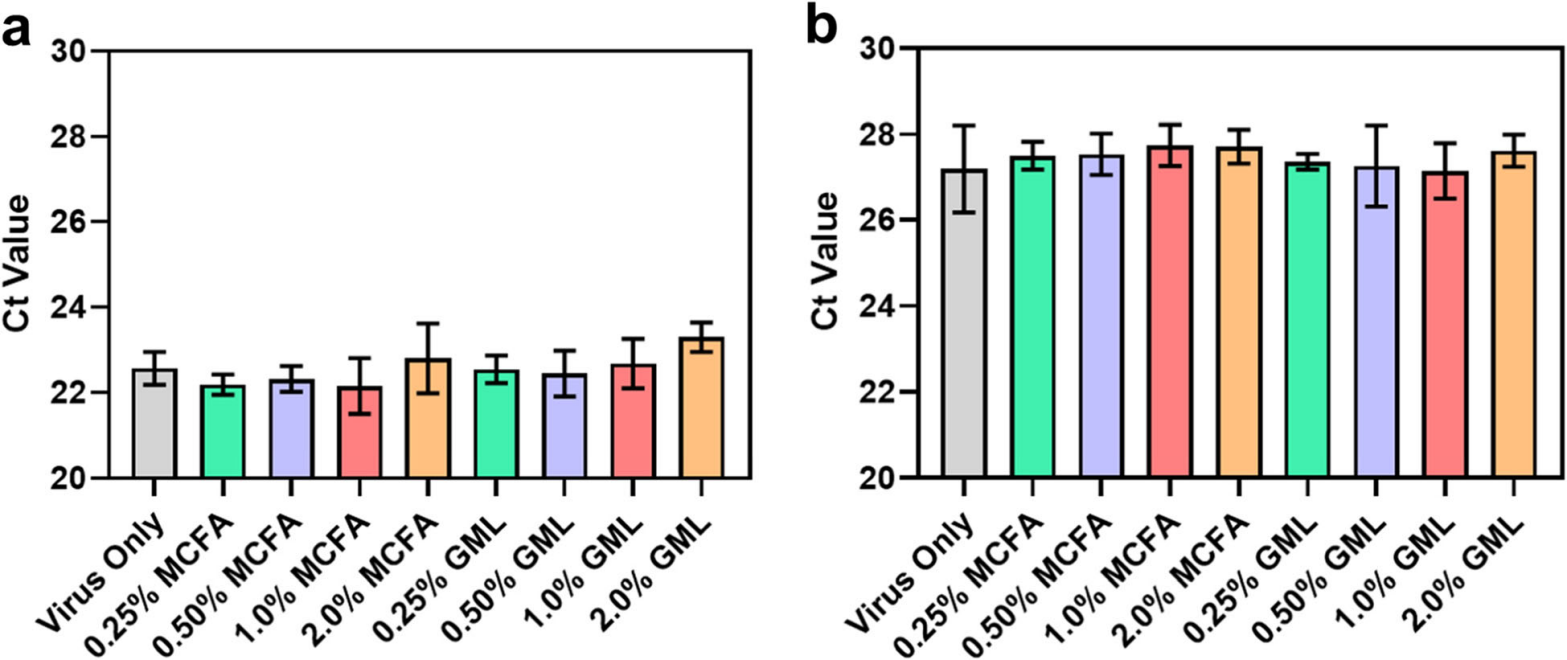
Effect of MCFA mixture and GML additives on ASFv genetic material in feed**.** 0–2.0% (wt/wt) feed additives were included in the feed and the effects on ASFv genetic material were measured (**a**) 30 min and (**b**) 24 h post-incubation. The amount of intact virus genetic material was determined by quantitative PCR assay. Data are reported as mean ± standard deviation from three independent experiments (*n* = 3 per group)

After 30-min incubation, the virus-only control and all MCFA-treated feed samples had Ct values in the range of 22 to 23 (Fig. 3a). This result indicated that the MCFA treatments did not affect virus genetic material. Likewise, the 0.25%, 0.50%, and 1.0% GML-treated feed samples had Ct values around 22 as well. On the other hand, the 2.0% GML-treated feed sample tended to have a larger Ct value around 23, which indicates a slight decrease in the amount of intact genetic material. On the other hand, the Ct values of the virus-only control and all MCFA- and GML-treated feed samples had increased to around 27 after 24-h incubation (Fig. 3b). This finding indicates that the virus genetic material modestly degraded in all test groups due to the storage conditions, while the MCFA and GML additives did not further affect the integrity of virus genetic material. Together with the viral titer measurements, these data support that GML abrogates ASFv infectivity in feed through a virucidal mechanism that impairs virus particle structure but does not directly damage virus genetic material.

### Effect on antibody recognition of viral antigens

While GML is a membrane-disruptive compound that is known to damage the lipid membrane surrounding enveloped viruses, we also sought to determine if GML treatment affected virus antigenicity in the feed samples. In general, ASFv particles have a five-layer structure that consists of an inner nucleoid containing virus genetic material, a core shell consisting of proteins, an inner lipid membrane coating, an icosahedral protein capsid, and an outer lipid membrane coating (“envelope”) [47]. The p72 protein is the major structural component of the viral capsid and also one of the key viral antigens [48, 49]. Therefore, we also conducted ELISA experiments on the ASFv-contaminated feed samples to determine if GML treatment affects antibody recognition of the antigenic p72 protein. A larger optical absorbance density (OD) signal indicates the presence of more structurally intact p72 protein and vice versa.

**Fig. 4:**
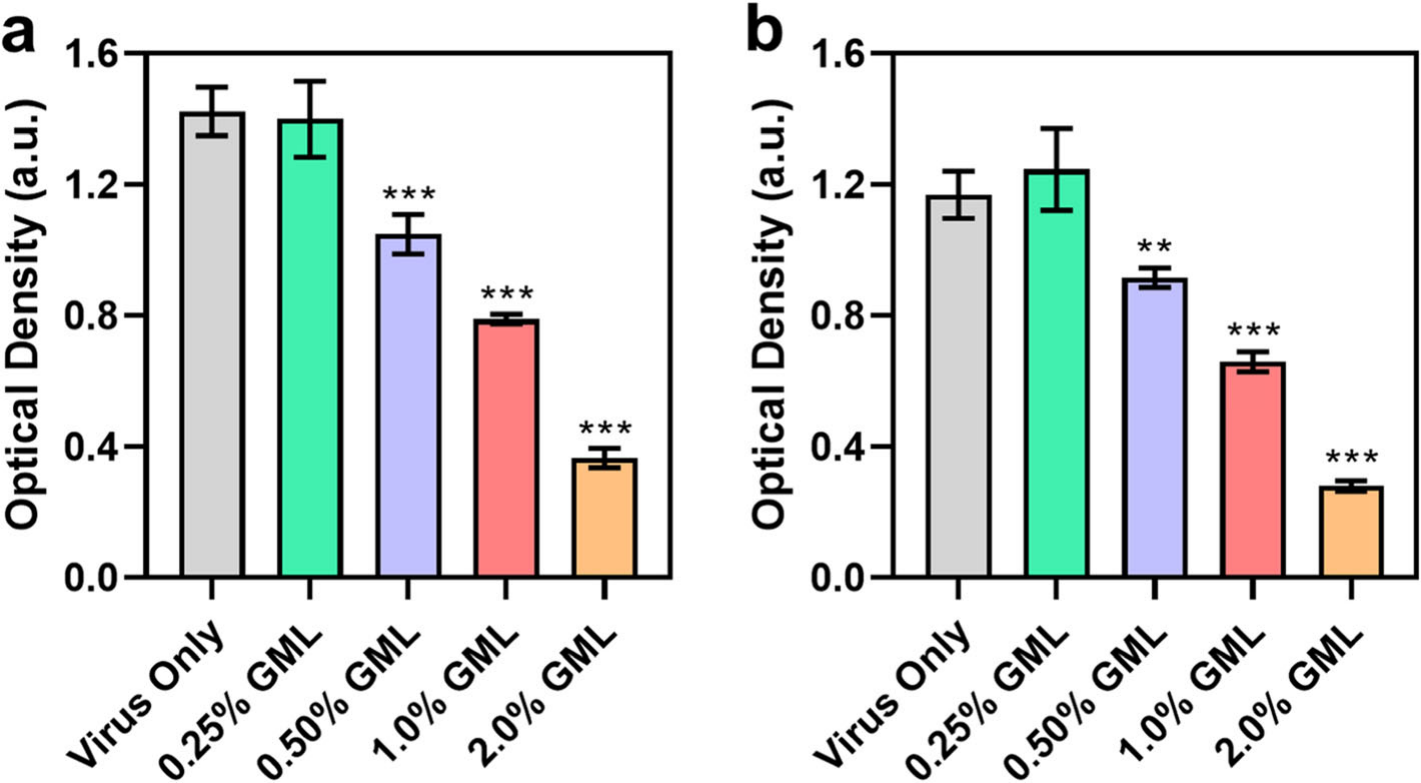
Effect of MCFA mixture and GML additives on ASFv antigen levels in feed. 0–2.0 (wt/wt) feed additives were included in the feed and the effects on ASFv antigen levels were measured (**a**) 30 min and (**b**) 24 h post-incubation. The relative amount of structurally intact p72 protein antigen was measured by ELISA. Data are reported as mean ± standard deviation from three independent experiments (*n* = 3 per group). The markers ** and *** indicate *P* < 0.01 and *P* < 0.001, respectively, versus the virus-only control

After 30-min incubation, the virus-only control had a mean OD value of 1.42 while sufficiently high doses of GML additive caused dose-dependent decreases in the signal intensity (*P* < 0.001) (Fig. 4a). There was no change in the 0.25% GML-treated feed sample, while the 0.50%, 1.0%, and 2.0% GML-treated feed samples had significantly decreased p72 protein levels, as indicated by mean OD values of 1.05, 0.79, and 0.37, respectively. Similar results were observed after 24-h incubation, in which case the virus-only control had an OD value of 1.17% and 0.25% GML treatment did not change p72 protein levels (Fig. 4b). On the other hand, the OD values of the 0.50%, 1.0%, and 2.0% GML-treated feed samples were significantly decreased to 0.92, 0.66, and 0.28, respectively, indicating a dose-dependent response (*P* < 0.001). While the duration of feed storage had a modest effect on p72 protein levels, the dominant factor was the presence of GML additive and 0.50% and higher GML doses caused significant reductions at both time points.

These data support that GML treatment caused decreases in the amount of structurally intact p72 protein, which is possibly related to conformational changes in the structure of the epitope site detected in the ELISA measurements. Indeed, two recent cryogenic transmission electron microscopy studies [35, 50] have reported how p72 proteins interact with the inner lipid membrane in order to stabilize the capsid network. Conversely, GML-mediated membrane disruption likely affects the outer and/or inner lipid membranes of ASFv particles, and such effects would in turn destabilize the capsid network, including inducing conformational changes in p72 protein structure. This finding is supported by recent findings that showed cholesterol depletion of human immunodeficiency type 1 (HIV-1) virus particles – another form of viral membrane disruption – decreases the conformational stability of membrane-associated viral proteins [51]. Notably, the 0.50% and 1.0% GML treatments also caused significant drops in p72 protein levels even though those levels did not significantly affect ASFv infectivity levels as described above. This finding suggests that GML-mediated changes in virus particle structure precede drops in virus infectivity and a critical degree of structural changes is needed to abrogate infectivity. Taken together, our results demonstrate that GML inclusion helps to inhibit ASFv infectivity in feed by up to 98% within 24 h and GML-mediated membrane disruption likely affects not only the lipid bilayer membranes of ASFv particles but also related structural proteins that rely on those membranes for stabilizing support.

## Conclusions

In this study, the antiviral activities of selected MCFA and GML to inhibit ASFv *in vitro* were investigated and feed additive candidates were discovered that can significantly reduce ASFv infectivity in feed environments as well. Solution-phase virucidal assay experiments identified that all tested MCFA and GML inhibited ASFv at 5 mmol/L compound concentrations while more detailed investigations showed that only GML maintained a similar level of virucidal activity at 250 μmol/L compound concentration. Additional antiviral assay experiments showed that GML also inhibited ASFv via one or more other inhibitory mechanisms as well. Based on these results, an MCFA mixture and GML were tested as two feed additive candidates to inhibit ASFv infectivity in feed, as measured by infectious viral titer measurements. While the MCFA mixture did not significantly inhibit ASFv in feed, the GML additive exhibited dose-dependent inhibitory effects at 1.0% and higher GML inclusion rates and the 2.0% GML dose significantly reduced the amount of infectious virus in feed samples at both the 30 min and 24 h time points. Mechanistic studies further revealed that the antiviral activity of GML in feed caused conformational changes in viral membrane-associated ASFv p72 structural protein but did not affect viral genetic material, which is consistent with the known membrane-disruptive effects of GML. Looking forward, the findings support that MCFA and GML can inhibit ASFv *in vitro* and that GML is more potent than MCFA to inhibit ASFv in liquid conditions on account of exhibiting antiviral activity at a lower compound concentration. Importantly, the data also support that GML as a feed additive can reduce ASFv infectivity in feed environments. Further exploration of additional MCFA mixtures alone or together with GML and other monoglycerides is warranted, especially since recent findings show that another carrier-free MCFA mixture can inhibit ASFv in feed [52] and that MCFA and GML blends can exhibit synergistic phospholipid membrane-disruptive activities [53]. Continued development of suitable formulations to maximize the antiviral performance and industrial utility of MCFA and GML for water and feed delivery [54] could help these additives become important tools in combating the infection and spread of swine viral pathogens, including ASFv.

## Supplementary information

Additional file 1. **Figure S1.** Dose-dependent evaluation of GML to inhibit ASFv infectivity in antiviral assay. **Figure S2.** Evaluation of different MCFA and GML to inhibit ASFv infectivity in antiviral assay at 250 μmol/L compound concentrations. (DOCX 23 kb)

## Data Availability

The datasets used and/or analyzed during the current study are available from the corresponding authors on reasonable request.

## Abbreviations

ASFv: African swine fever virus
CPE: Cytopathic effect
EMEM: Eagle’s minimum essential medium
ELISA: Enzyme-linked immunosorbent assay
FBS: Fetal bovine serum
GML: Glycerol monolaurate
MCFA: Medium-chain fatty acids
PBS: Phosphate-buffered saline
PCR: Polymerase chain reaction
TCID_50_: 50% tissue culture infective dose
