# Supplementary material for: Inhibition of African swine fever virus in liquid and feed by medium-chain fatty acids and glycerol monolaurate
Source: J Anim Sci Biotechnol. 2020 Dec 8;11:114. doi: 10.1186/s40104-020-00517-3 (PMC7722453; doi:10.1186/s40104-020-00517-3)
Supplement: Supplementary file 1 — Figure S1. Dose-dependent evaluation of GML to inhibit ASFv infectivity in antiviral assay. Figure S2. Evaluation of different MCFA and GML to inhibit ASFv infectivity in antiviral assay at 250 μmol/L compound concentrations. (DOCX 23 kb) [file 40104_2020_517_MOESM1_ESM.docx]

**Fig. S1.** **Dose-dependent evaluation of GML to inhibit ASFv infectivity in antiviral assay.** The tested GML concentrations were 16, 31, 63, 125, and 250 μmol/L along with a virus-only control (denoted as 0 μmol/L GML). The data corresponding to 0 and 250 μmol/L GML are replotted from Fig. 1c and all data were collected in the same experimental series. Viral titers were determined by CPE-based assay. Data are reported as mean ± standard deviation from three independent experiments (*n* = 3 per group). The markers * and *** indicate *P*<0.05 and *P*<0.001, respectively, versus the virus-only control.

**Fig. S2.** **Evaluation of different MCFA and GML to inhibit ASFv infectivity in antiviral assay at 250 μmol/L compound concentrations.** The tested compound concentrations were 250 μmol/L along with a virus-only control. The data corresponding to the virus-only control and 250 μmol/L GML are replotted from Fig. 1c and all data were collected in the same experimental series. Viral titers were determined by CPE-based assay. Data are reported as mean ± standard deviation from three independent experiments (*n* = 3 per group). The marker *** indicates *P*<0.001 versus the virus-only control. One-way ANOVA with Tukey’s multiple comparisons test was also performed and showed that the GML treatment led to a more significant drop in viral titer compared to each of the MCFA treatment groups (*P*<0.001).
